# Actinomyces Species As Emerging Pathogens: An Observational Study of Clinical Infections and Microbiological Implications

**DOI:** 10.7759/cureus.77128

**Published:** 2025-01-08

**Authors:** Abraham A Ayantunde, Joanne Kiang, Nadeem S Raja, Javeed Ahmed, Anjali Sanghera, Saumya Venkatesha, Andrew C Ekwesianya

**Affiliations:** 1 Department of General Surgery, Southend University Hospital, Southend-on-Sea, GBR; 2 Department of Surgery, Southend University Hospital, Southend-on-Sea, GBR; 3 Department of Microbiology, Medway and Maritime Hospital NHS Trust, Gillingham, GBR; 4 Department of Clinical Microbiology, Southend University Hospital, Southend-on-Sea, GBR; 5 Department of General and Colorectal Surgery, Southend University Hospital, Southend-on-Sea, GBR

**Keywords:** actinomyces species, bacteraemia, culture, empirical antibiotics, gram-positive, infections, microbiology, polymicrobial

## Abstract

Introduction: *Actinomyces *species, Gram-positive filamentous anaerobic microaerophilic organisms, are commensals of the human oropharynx, gastrointestinal, and urogenital tracts. Actinomycosis is rare and occurs when tissue integrity is compromised, typically in a polymicrobial fashion. There is an emerging rise in *Actinomyces *species-associated infections, with attendant therapeutic challenges.

Aim: We evaluated the pattern, presentation, and risk factors for *Actinomyces *species-associated infections.

Material and methods: Blood culture, tissue, fluids, bone, and swab samples with isolated *Actinomyces *species were evaluated between July 2016 and April 2021. The antibiotic susceptibility of the isolated *Actinomyces *was obtained as per the European Committee on Antimicrobial Susceptibility Testing (EUCAST) guidelines. Electronic medical records were retrospectively evaluated for demographic and clinicopathological data relating to the patients.

Results: A total of 145 patients were evaluated, comprising 63 males and 82 females; the mean age was 49 years. About 52.4% and 59.3% of comorbidities and risk factors for *Actinomyces *species infection susceptibility, respectively. The most common presentations were infected sebaceous cysts (25.5%) and pilonidal abscesses (13.8%). *Actinomyces *species were isolated from swabs (78), pus (32), blood cultures (26), body fluids (6), soft tissues (2), and bone fragments (1). Eleven different *Actinomyces *species were isolated, and commonly isolated species were *Actinomyces neuii *(24.8%), *Actinomyces turicensis *(22.8%), and *Actinomyces europaeus *(13.8%). About 57.2% of the samples had mixed microorganisms isolated; 26 of 29 blood culture samples yielded *Actinomyces, *and 6 of 29 yielded mixed microbial agents. A majority (78.6%) of the patients received empirical antibiotics, and 79% of the antibiotic choice was appropriate.

Conclusion​​​​​: *Actinomyces *species isolates and infections are increasingly reported, potentially attributed to improved culture techniques. We recommend epidemiology and resistance surveillance in *Actinomyces *species-associated infections.

## Introduction

*Actinomyces *species infection is a rare type of infection caused by Gram-positive filamentous anaerobic microaerophilic bacteria from the Actinomycetaceae family (genus *Actinomyces*) [1]. They are rod-shaped, pigment-producing bacteria that form branching filaments resembling those of fungi and are generally commensals of the human oropharynx and gastrointestinal and female urogenital tracts [1,2]. Colonization is common in healthy individuals, but *Actinomyces *is associated with a wide range of infections in people with poor oral hygiene and dental caries, immunosuppression, and local tissue disruption predisposing to abscesses and suppurative intra-abdominal and pelvic infections, as well as bacteremia [1]. Actinomycosis as a disease entity is more common in females than males, of course, except in those associated with intrauterine contraceptive devices (IUCDs) and pelvic inflammatory disease [1]. There are more than 30 species of *Actinomyces *that have been described in the literature [1-4]. Many species of *Actinomyces *have been isolated that are associated with pathogenic presentations specific to particular anatomical sites, but the commonly encountered subtype in human actinomycosis is *Actinomyces israelii *[1-4].

Actinomycosis occurs when there is a compromise of tissue integrity, such as a breach in the mucosal protection of their habitat or the presence of soft tissue necrosis, thereby becoming pathogenic and resulting in granulomatous changes, formation of abscess, tissue destruction, or fistulation [1-4]. *Actinomyces *species acquire their pathogenicity through the invasion of breached or necrotic tissues. Infection by *Actinomyces *species is therefore considered endogenous [1].

*Actinomyces *species are often isolated in cultures with other commensals or other pathogenic microorganisms, either in a co-infection relationship or as opportunistic colonizers [1-4]. Therefore, *Actinomyces *infection usually occurs in a polymicrobial fashion in association with other companion mixed microorganisms. The isolates may include several bacterial species, and it is believed that human infection requires the presence of such associated bacteria with the production of toxins and enzymes, which encourage the inhibition of the host defense system and contribute to tissue destruction [1,3-6]. It is also thought that the polymicrobial invasion converts the aerobic microenvironment into an anaerobic one, in which *Actinomyces *species thrive to infect the damaged tissues [1-5]. *Actinomyces *species are often present and isolated with other organisms such as *Aggregatibacter actinomycetemcomitans*, *Eikenella corrodens*, Capnocytophaga, Fusobacteria, Bacteroids, Staphylococci, Streptococci, and Enterobacteriaceae, depending on the site of infection [3-6].

Increasingly, *Actinomyces *species are associated with a wide range of infections at different anatomical sites of the body. There seems to be an emerging rise in the microbiological sample specimens with isolated *Actinomyces *species, and this phenomenon has been observed over the last few years in our microbiology department. Interestingly, over the last few years, there has been a rise in the number of reported blood cultures positive for *Actinomyces *species. Improved modern laboratory diagnostic techniques, increased awareness among microbiology staff and clinical teams, and the use of specific growth media for *Actinomyces *have facilitated its successful cultivation from clinical samples. It is also postulated that *Actinomyces *species isolated in these microbiological samples may be due to colonization.

The aim of this observational study was to evaluate the pattern and characteristics of the *Actinomyces *species associated with infections of various anatomical sites and to assess the risk factors and mixed microbes involved in the co-infection.

## Materials and methods

We conducted a retrospective study, from July 2016 to April 2021, on patients from the South Essex area of England covered by Southend University Hospital (SUH) and Basildon University Teaching Hospital (BUTH), the two major teaching hospitals with a total bed capacity of 1500. Both hospitals provide healthcare services to a population of about 800,000 people in the South Essex area.

Patient selection

A total of 145 patients with suspected or identifiable sources of infection containing *Actinomyces *species were identified from the prospectively maintained microbiology database and information system over the study period. Patients who had presented with signs and symptoms of infection were included in this study if they had *Actinomyces* species isolated from their clinical specimens such as wound swabs, blood culture, pus, bone, and/or other tissue samples.

Patient electronic records were reviewed to collect data including age, gender, site of infection and/or clinical diagnosis, underlying illness, risk factors, and vital signs, including Modified Early Warning (MEW) Score. Laboratory parameters such as C-reactive protein (CRP), white blood cell (WBC) count, neutrophil count, the nature of the specimen, species of isolated *Actinomyces*, other associated micro-organism isolates, and antibiotic susceptibility of the clinical isolates were also recorded.

Microbiological analysis

Clinical samples of blood cultures, tissues from wounds, needle aspirates of pus, bone biopsies, and wound swabs were taken from patients with symptoms and signs of infection and sent to the clinical microbiology laboratory. The blood culture set was ordered by the attending physician in case of systemic illness. Blood samples were introduced into aerobic and anaerobic bottles of the blood culture set and loaded into an automated blood culture system known as BacT/ALERT (Biomerieux, Durham, USA). These bottles were incubated at 37°C for five days. All bottles showing bacterial growth were subcultured onto nutrient, chocolate, or blood disc agar plates containing FAA and 5 µg of metronidazole.

Other samples were placed in sterile universal containers. All specimens were inoculated on the above-mentioned agar plates. The plates were incubated in aerobic and anaerobic conditions. Isolated anaerobic organisms were further identified by the conventional and automated method systems such as API, VITEK II (bioMérieux, France), and MALDI-TOF MS (Bruker Daltonik, Bremen, Germany).

Antimicrobial sensitivity testing

Antibiotic susceptibility testing on *Actinomyces *by disc susceptibility method was in accordance with The European Committee on Antimicrobial Susceptibility Testing (EUCAST), and the results were interpreted according to EUCAST guidelines [7]. Antibiotic sensitivity was done using clindamycin, amoxicillin/clavulanic acid, metronidazole, penicillin/tazobactam, and meropenem discs, and E-test strips were used on Muller-Hinton agar plates where indicated to determine the antibiotic sensitivity of anaerobes (EUCAST).

Statistical analysis

Data analysis was performed using IBM SPSS Statistics for Windows, Version 26 (Released 2019; IBM Corp., Armonk, New York, United States). Continuous and normally distributed variables were expressed in descriptive statistics as mean ± standard deviation (SD) and/or median, while categorical variables were presented as figures and/or percentages represented in tables and charts.

## Results

A total of 145 patients were included in the study, and their data were analyzed. The mean age was 49 ± 20 (SD) years. There were 63 males and 82 females, with an approximate gender ratio of 2:3. More than half (52.4%) of the study population had associated comorbidities at the time of presentation with infection. The distribution of the comorbidities is shown in Table 1.

**Table 1:** Distribution of associated comorbidities

Comorbidities	Number (N)	Percentage (%)
No comorbidity	69	47.6
Asthma	7	4.8
Diabetes mellitus	24	16.6
Hypertension	13	9.0
Ischemic heart disease	1	0.7
Congestive heart failure	2	1.4
Stroke	1	0.7
Psoriasis	1	0.7
Peripheral vascular disease	1	0.7
Active cancer	7	4.8
Chronic obstructive pulmonary disease	2	1.4
Inflammatory bowel disease	1	0.7
Hypothyroidism	4	2.8
Chronic liver disease	4	2.8
Epilepsy	2	1.4
Morbid obesity	1	0.7
Chronic liver disease	1	0.7
Intravenous drug abuser	3	2.1
Rheumatoid arthritis	1	0.7
Total	145	100.0

The median Carlson Comorbidity Index (CCI) was 1 (0-4). Risk factors for susceptibility to *Actinomyces *species infection were identified in 59.4% of the patients. Table 2 shows the distribution of the identified risk factors for *Actinomyces *species infections.

**Table 2:** Distribution of identified risk factors for Actinomyces species infection

Risk factors	Number (N)	Percentage (%)
No risk factor identified	59	40.6
Diabetes mellitus	25	17.2
Chronic smoking	19	13.1
Presence of malignancy ± chemotherapy	12	8.3
Recent abdominal surgery/intra-abdominal sepsis	6	4.1
Chronic liver disease	4	2.8
Intravenous drug abuse	2	1.4
Steroid use	8	5.5
Morbid obesity	1	0.7
Chronic kidney disease	1	0.7
Congestive heart disease	4	2.8
Poor oral hygiene and/or recent dental treatment	2	1.4
Presence of intra-uterine contraceptive device (IUCD)	1	0.7
Immunosuppression	1	0.7
Total	145	100

Patients with *Actinomyces *species-proven infections presented with a wide range of site-specific infections. As shown in Table 3, infected sebaceous cysts at different body sites were the commonest presentation in about 25.5% of the patients, and this was followed by pilonidal abscess in 13.8%.

**Table 3:** Distribution of site-specific presenting diagnoses

Diagnosis	Number (N)	Percentage (%)
Breast abscess	12	8.3
Buttock abscess	13	9.0
Labial abscess	3	2.1
Infected sebaceous cysts	37	25.5
Perianal abscess	7	4.8
Pilonidal abscess	20	13.8
Groin abscess	8	5.5
Abdominal wall abscess	4	2.8
Infected abdominal wall surgical wound	5	3.4
Intra-abdominal sepsis	9	6.2
Pelvic inflammatory disease	2	1.4
Infected orthopedic implants	3	2.1
Head and neck infection	6	4.1
Urinary tract infection	4	2.8
Septicemia (Blood-stream infection)	6	4.1
Chest infection	6	4.1
Total	145	100.0

The Modified Early Warning Score (MEWS) combines a patient's vital signs into a single aggregated score to assess their clinical status. It is calculated by evaluating key components, including heart rate, temperature, blood pressure, respiratory rate, and level of consciousness. High temperature, tachycardia, hypotension, tachypnea, and high MEW scores were present in 18.6%, 33.1%, 4.1%, 14.5%, and 43.4% of the patients, respectively. The presenting vital signs are represented in Table 4.

**Table 4:** Distribution of the presenting vital signs MEW: Modified Early Warning

Vital signs parameters	Patients with normal values (percentage)	Patients with abnormal values (percentage)
Temperature	118 (81.4%)	27 (18.6%)
Heart rate	97 (66.9%)	48 (33.1%)
Blood pressure	139 (95.9%)	6 (4.1%)
Respiratory rate	124 (85.5%)	21 (14.5%)
MEW score	82 (56.6%)	63 (43.3%)

The distribution of inflammatory markers in routine laboratory blood results associated with the identified infections is shown in Table 5.

**Table 5:** Distribution of blood inflammatory markers associated with the infections

Laboratory parameters	Patients with normal values	Patients with abnormal values
Hemoglobin levels	120	25 (low)
Total white blood cells	77	68 (elevated)
Neutrophils	91	54 (elevated)
C-reactive protein levels	37	108 (elevated)

There were 11 different species of *Actinomyces *organisms isolated from the studied samples, including a group that was not fully characterized. The distribution of these isolated *Actinomyces *species is shown in Table 6.

**Table 6:** Distribution of isolated Actinomyces species in the patients

*Actinomyces *species	Number (N)	Percentage (%)
Actinomyces europaeus	20	13.8
Actinomyces funkei	9	6.2
Actinomyces hominis	1	0.7
Actinomyces israelii	3	2.1
Actinomyces meyeri	3	2.1
Actinomyces naeslundii	3	2.1
Actinomyces neuii	36	24.8
Actinomyces odontolyticus	7	4.8
Actinomyces turicensis	33	22.8
Actinomyces urogenitalis	5	3.4
*Actinomyces *species (uncharacterized)	25	17.2
Total	145	100.0

About 57.2% of the patients had other mixed micro-organisms isolated from the samples in addition to the *Actinomyces *species. In 29 patients (20%) who had blood culture done in addition to other site-specific microbiology samples, *Actinomyces *was isolated in 23 blood culture specimens and other mixed micro-organisms in two blood culture samples. Table 7 shows the distribution of other site-specific samples sent with the blood culture.

**Table 7:** Distribution of site-specific samples sent with the blood culture

Site-specific samples	Number (N)	Percentage (%)
Sputum	7	4.8
Pelvic abscess and IUCD	4	2.8
Infected orthopedic implants	3	2.0
Catheter tips in chemotherapy patients	4	2.8
Necrotizing fasciitis tissues	1	0.7
Infected sebaceous cyst	1	0.7
Urine samples	3	2.0
Liver abscess	1	0.7
Leg abscess	1	0.7
Puerperal sample	1	0.7
Abscess from perforated rectosigmoid cancer	1	0.7
Perianal abscess	1	0.7
Pilonidal abscess	1	0.7
Total	29	20.0

Surgical infections were the most common source of *Actinomyces *species isolated. Surgical interventions were offered to 79.3% of the patients while only 20.7% had antibiotic treatment alone. Empirical antibiotics were administered in 78.6% of the patients and the choice was found to be appropriately selected in 79.0%.

## Discussion

This observational study evaluated a cohort of patients with symptoms of *Actinomyces *species-associated infections at various body sites. The presenting sites of the infections included deep, superficial cutaneous, and subcutaneous suppurations. There seems to be an increasing trend in the isolation of *Actinomyces *species from microbiological samples from patients presenting with different body site infections [1-3]. The published literature contains many reports of site-specific anatomical presentations of actinomycosis, such as oral, cervicofacial, pulmonary, and abdominopelvic infections with acute to chronic granulomatous presentations [1-3,8-12]. However, the literature is sparse with various site-associated *Actinomyces *species in deep and superficial soft tissue infections. Reports of *Actinomyces *bacteremia are even rarer, but the pickup rate of such manifestations is also on the rise because of the increased availability of advanced microbiological diagnostic techniques [1-4,8-12].

We have studied and presented the results of this cohort population to highlight the miscellaneous clinical presentations and characteristics of *Actinomyces *species-associated infections. Isolation of *Actinomyces *species in any microbiological samples from patients presenting with any site infections must be considered significant, and an appropriate treatment strategy instituted. Also important is the observation that common surgical infections, especially soft tissue abscesses, were the most common source of *Actinomyces *species isolated in the samples.

The literature attested to the fact that many patients presenting with *Actinomyces *species-associated infections have at least one immunosuppressive risk factor encouraging tissue invasion of the organisms. The identified risk factors that have been predominantly reported include patients aged >65 years, malignancies, HIV infection, obesity, diabetes mellitus, chronic alcoholism, prolonged steroid use, women with intrauterine devices, and iatrogenic immunosuppression [8,9,11,13-15]. However, other authors have reported the absence of any predisposing risk factors in their patients in the published literature [1-3,15]. We have identified susceptibility risk factors in 59.3% of our patients in this study, including diabetes mellitus, presence of malignancies, use of steroids, IUCD, intravenous drug abuse, recent abdominal and/or pelvic surgery, and chronic kidney/liver/cardiac disease.

There was a significant number of patients (40.6%) in the studied cohort with no identifiable predisposing factors. Chen et al. have reported splenic actinomycosis abscess in a patient with acute myeloid leukemia as the predisposing risk factor [16]. Fiorino, in a systematic review, also reported on publications identifying IUCD as the predisposing risk factor in the causation of actinomycotic abscess and pelvic inflammatory disease (PID) [17]. Steininger and Willinger reported cases of *Actinomyces *and *Norcadia *infections in both immunocompromised and immunocompetent patients in equal measure [18]. Intrauterine contraceptive devices (IUCDs) have been found to be a significant risk factor in women and associated with *Actinomyces *species PID and pelvic abscesses.

*Actinomyces *species infections typically occur when there is tissue damage to anatomical barriers, and this is generally achieved with co-infection with other mixed microorganisms, which contribute to the pathogenesis of the actinomycotic infection by inhibiting the host defenses [1-6]. The majority of *Actinomyces* species-associated infections are polymicrobial with multiple organisms isolated along with the bacteria. About 57.2% of our patients had mixed microorganisms isolated in association with *Actinomyces *species. Our finding is consistent with what has been reported in the literature. Bonnefond et al. [2] reported a co-infection with mixed microorganisms in 46% of their patients, while Könönen and Wade [3] found the proportion of polymicrobial association in *Actinomyces *species-associated infections to be as high as 75-95%. Pathogenesis of *Actinomyces *species-associated infection occurs in a polymicrobial fashion, which contributes to the pathogenic process by synergistically enhancing the infection process [2-5].

The commonest sites of *Actinomyces *species infections reported in this study were cutaneous and subcutaneous soft tissue suppurations, accounting for 76.6% of the presentations. The remaining 23.4% of the patients presented with deep-seated infections. There were 45 patients with buttock, perianal, pilonidal, and groin abscesses in this cohort, but none of them typically presented with a multi-fistulating disease that has been widely reported in the literature with actinomycosis. The majority of these patients had associated mixed anaerobes in addition to the *Actinomyces *species and did not present with multiple fistulations. Dosis et al. in a review of the literature on perianal actinomycosis concluded that perianal actinomycosis is rare, with case series and isolated case reports published [19].

Our study demonstrated that all cases of *Actinomyces *species were confirmed from cultures taken from various anatomical sites in patients presenting with symptoms. The introduction of matrix-assisted laser desorption-ionization time-of-flight mass spectrometry (MALDI-TOF MS) has represented a technological revolution in microbiology diagnostics, which has allowed for fast, accurate, and reliable identification of anaerobic organisms, including *Actinomyces *species [20,21]. Only two of our patients had soft tissue samples sent for microbiological and histological confirmation of *Actinomyces *species. One patient had a sample of bone fragments sent for microbiological confirmation. These infections do not represent typical actinomycosis but were considered to be simple *Actinomyces *species-associated infections with various body site suppurations. Tissue culture and/or histology with evidence of sulfur granules is the most significant confirmatory diagnostic process of *Actinomyces *species infection [1-4,19].

We isolated 11 different species of *Actinomyces *organisms from the studied population, including a group that was not fully characterized into a particular species level. The most isolated species that has been found to be associated with pathogenic presentations of classical human actinomycosis in the literature is *A. israelii *[1-6]. However, in the current cohort of *Actinomyces *species-associated infections, the commonest isolated species were *Actinomyces neuii*, *Actinomyces turicensis*, and *Actinomyces europaeus,* accounting for 61.4% of the overall *Actinomyces *species isolates in the cohort. Our study population showed that the commonest causes of bacteremia were *Actinomyces odonlolyticus*, *A. turicensis*, and *A. neuii*. Mollet and Marek, in their report of *Actinomyces *species bacteremia and associated one-year mortality, demonstrated that *Actinomyces viscous *was the most isolated offending organism, accounting for 47% of their cohort [8]. An underlying malignancy was the commonest susceptibility risk factor for *Actinomyces *species bacteremia. Mollet and Malek also attested to the fact that underlying hematological and solid organ malignancies were significant risk factors in their patient population [8]. Ali et al. have also reported 15 patients of *Actinomyces *bacteremia with *Actinomyces odontolyticus* with a related mortality of 6.6% [11].

*Actinomyces *species-associated infections are treatable, as the organisms are generally susceptible to most of the β-lactam antibiotics [2]. In our study, no multidrug-resistant organism was isolated, and no specific antibiotic sensitivity was linked to any species. Smith et al., in a study of 87 cases of *Actinomyces *species isolates, demonstrated that most of these species were highly susceptible to β-lactam agents such as benzylpenicillin, amoxicillin, ceftriaxone, meropenem, and piperacillin-tazobactam. *Actinomyces *species identification and antibiotic susceptibility were performed according to the European Committee on Antimicrobial Susceptibility Testing, and the results were interpreted according to EUCAST guidelines [7]. There is currently no standardized consensus or guidelines for the choice of antibiotic treatment for *Actinomyces *species, as most recommendations are based on case series and in vivo studies [2,18,19,22]. Long-term antibiotic treatment is generally recommended for typical actinomycosis [1-4,23]. The duration of antibiotic treatment is variable in published literature, and some authors have even advocated a shorter duration of therapy [1,2,22-24]. There is no agreed consensus on the duration of antibiotic therapy for *Actinomyces *species-associated infections reported in this study. There was also no record of any treatment failure or repeat sampling among the patients. No chronicity or seeding to other parts of the body was reported.

The initial empirical antibiotics were administered in 78.6% of the patients before the microbiology culture results were available, and the choice was found to be appropriately selected in 79.0% of the patients. Empirical antibiotic choice consisted of co-amoxiclav, azithromycin, cetriaxone, clindamycin, erythromycin, flucloxacillin, metronidazole, piperazine, tazocin, teicoplanin, clarithromycin, and co-trimoxazole as single agents and in various combinations. Surgical interventions were offered to 79.3% of the patients in addition to antibiotic therapy, while 20.7% had antibiotic treatment alone.

One major limitation of our study is that, being retrospective, we were not able to have accurate data on the duration of antibiotic treatment for all our patients. Many patients who were considered clinically stable were treated on an outpatient basis in the community. The other limitation of the study is the lack of robust follow-up data on all our patients. There was a subset of *Actinomyces *species that was not fully characterized by our isolation and culture techniques and therefore could not be appropriately classified into any of the existing species. This phenomenon has been previously described [2,3,9].

## Conclusions

*Actinomyces *species-associated infection and bacteremia, previously described to be rare, are now increasingly and commonly reported in the cultures of microbiological samples due to improved and sensitive culture techniques. The ready availability of new techniques of isolation hopefully will give rise to early diagnosis, prompt treatment, and better outcomes for patients. Microscopic findings of necrosis with yellow sulfur granules and filamentous Gram-positive fungal-like pathogens with prolonged microbiology culture under anaerobic conditions are necessary for the identification of *Actinomyces *species.

We have identified specific susceptibility risk factors for *Actinomyces *species-associated infections in this study, which should be of interest to our colleagues. The typical fistulating disease was not identified in this study. All clinicians need to be aware of the rising tide of isolation of *Actinomyces *species from microbiological samples and the need for this to be considered significant in patients presenting with symptoms and signs of infection.
